# Acetozone in Gastrointestinal Disease

**Published:** 1903-04

**Authors:** W. F. Drake

**Affiliations:** New York


### CLINICAL REPORT.

### Acetozone in Gastrointestinal Disease.

By W. F. DRAKE, M. D., New York.

CASE I.—A. B., 56 years of age, was brought to me suffering from gastrointestinal catarrh and chronic colitis, which had lasted for six years. For a long time he had been treated with high enemas of salol, resorcin, and the like, but without benefit. Finally, I determined to try acetozone, of which I gave him a quantity of the 1-1000 solution after meals, with instructions that the colon should be irrigated daily with normal salt solution. Under this treatment a marked improvement was manifested in a few days; the flatulency disappeared and the patient suffered absolutely no pain. Prior to the exhibition of acetozone his heart's action was very irregular, but it now became perfectly normal. The acetozone treatment was continued for about six months when the patient was discharged. He still continues to use enemas of salt solution, once a week, and takes a dose of cascara evacuant as a laxative, occasionally. None of the former distressing symptoms have returned.

Case II.—C. D., 34 years of age, after suffering from acute catarrhal appendicitis, for which the usual treatment of ice bags and high enemas had been employed, made a fairly good recovery. He afterward suffered from intestinal fermentation, accompanied by flatulency, tenderness in the region of the appendix, very irregular heart's action, mitral regurgitation, severe sick headache, foul breath, and a heavily coated tongue. I prescribed 30 drops of cascara evacuant each evening as a laxative, and the acetozone solution after meals. After three months of this treatment the patient was discharged cured.

